# Anaemia Associated with Trypanosomes Infections in Cattle of West Gojjam Zone, Northwest Ethiopia

**DOI:** 10.1155/2021/5531537

**Published:** 2021-06-29

**Authors:** Kumela Lelisa, Behablom Meharenet

**Affiliations:** National Institute for the Control and Eradication of Tsetse Fly and Trypanosomosis, Akaki Kaliti Sub-City P.O. Box 19917, Addis Ababa, Ethiopia

## Abstract

**Background:**

African animal trypanosomosis is a major veterinary problem over a large area of the tsetse belt region of Africa. Anaemia is a cardinal sign of trypanosome infections. The mechanism of anaemia due to trypanosomosis is complex and multifactorial in origin. Packed cell volume (PCV) usually gives an indication of the anaemia and disease status of a trypanosome-infected animal.

**Methods:**

A cross-sectional study was conducted from December 2017 to January 2018 in West Gojjam zone, Northwest Ethiopia, to determine the trypanosome infections rate and the possible correlation between parasitic infection and anaemia using the dark ground buffy coat technique, Giemsa-stained thin blood smear, and PCV reading on a haematocrit reader.

**Results:**

The overall trypanosomosis prevalence was 7.81%, 95% CI = 7.45–8.17. *Trypanosoma congolense* (4.25%) and *T. vivax* (3.56%) were the trypanosomes species identified in the studied area. PCV for all sampled cattle was analysed to estimate the degree of anaemia. From the total examined animals (*N* = 730), 356 (48.77%) were anaemic and 374 (51.23%) were nonanaemic. The mean PCV of parasitemic cattle was significantly lower (21.09%, 95% CI = 20.13–22.05) than that of aparasitemic ones (25.96%, 95% CI = 25.68–26.24). There was a positive association between trypanosome infection and anaemia. Although both trypanosome species are significantly associated with a decreased herd mean PCV (<24), the mean PCV of cattle infected with *T. congolense* (19.45%) was lower than that of infected with *T. vivax* (23.04%). The herd mean PCV was not significantly associated to locations, age, and sex of the studied animals.

**Conclusions:**

The study confirms that the prevalence of trypanosomes infections and herd mean PCV has a significant association. The mean herd PCV can be a useful cheap tool to screen for possible trypanosome infection. However, there were cattle positive for trypanosomes having mean PCV within the reference interval and negative animals with anaemia. Furthermore, PCV reading should be confirmed by other diagnostic techniques to accurately conclude that trypanosomosis is the only cause of anaemia.

## 1. Introduction

African animal trypanosomosis is a major veterinary problem over a large area of the tsetse belt region of Africa. It is a chronically debilitating protozoan disease of livestock, which is of great economic importance in sub-Saharan Africa. Six trypanosomes species were reported in Ethiopia, although the vascular trypanosomes *T. congolense* and *T. vivax* are the most pathogenic, economically very important, and widely distributed in the country [1, 2].

The trypanosomes that cause this disease are extracellular protozoan parasites that have developed efficient immune escape mechanisms to manipulate the entire host immune response to allow parasite survival and transmission. Anaemia is a cardinal sign of trypanosome infections [3]. The mechanism of anaemia due to trypanosomosis is complex and multifactorial in origin [4]. The rate at which anaemia develops is influenced by energy intakes and protein gain [5].

The interplay of several factors acting either individually or synergistically contributes to the development of haemolytic anaemia in human and animal trypanosomosis. Most common among these factors are increased intravascular red blood cells destruction caused by lashing action of trypanosome flagella, undulating pyrexia, platelet aggregation, toxins and metabolites from trypanosomes, lipid peroxidation, and malnutrition. Meanwhile, idiopathic serum and tumour necrosing factors are responsible for dyserythropoieses [6].

PCV usually gives an indication of the anaemia and disease status of a trypanosome-infected animal and is correlated with animal production and reproduction performance. Reports indicated that the prevalence of trypanosome infections and herd mean PCV has a relationship. PCV is expected to decrease with the increasing prevalence of trypanosomosis. Hence, the relationship between the prevalence of trypanosomes infections and herd mean PCV could be a useful tool in the management of trypanosomosis and planning of its control. However, this relationship has not been quantified in Northwest Ethiopia, specifically West Gojjam zone. Therefore, this study aimed at determining the relationship between trypanosomes infection rates and occurrence of anaemia and estimating the prevalence of bovine trypanosomosis in West Gojjam zone, Northwest Ethiopia.

## 2. Methods

### 2.1. Study Area Description

**Figure 1:**
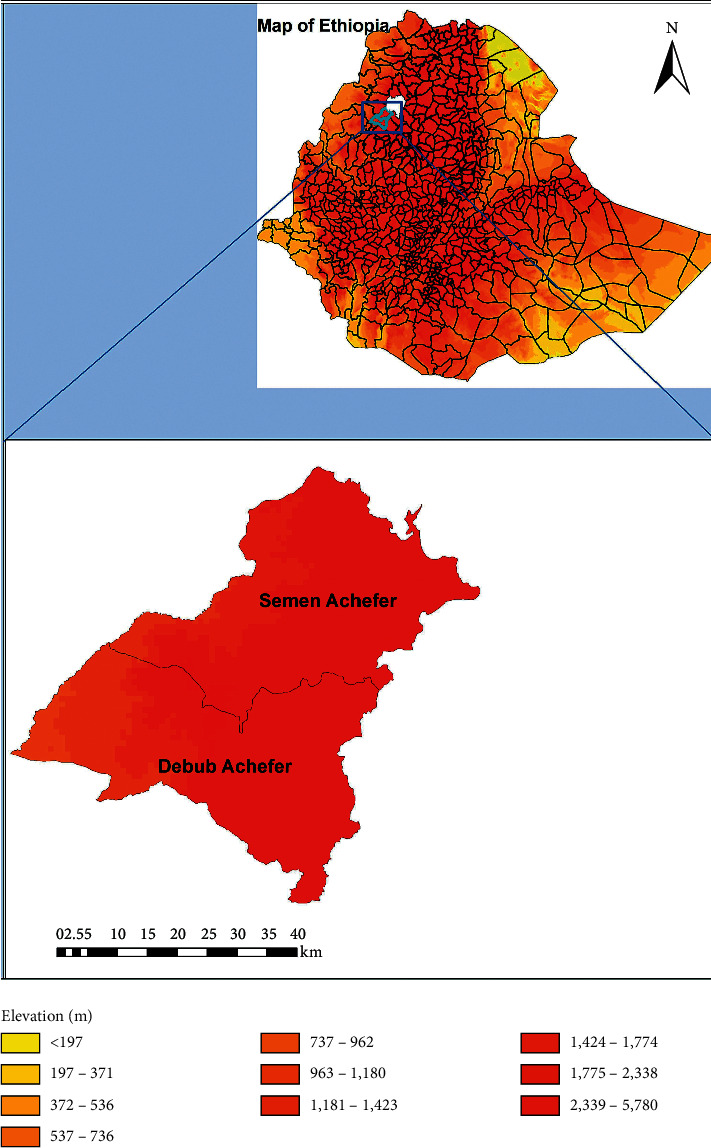
Map showing the geographical location of the study sites in Northwest Ethiopia.

West Gojjam zone is bordered on the south by the Abay River, which separates it from Oromia and Benishangul Gumuz regions, on the west by Awi zone, on the northwest by Northern Gondar, on the north by Lake Tana and the Abay River that separates it from South Gondar, and on the east by East Gojjam. The study was conducted in Debub Achefer and Semen Achefer (Figure 1). The area of the zone is 13,312 square kilometres.

The climate of the area is tropical. The annual mean temperature in West Gojjam ranges 12.9–29.5°C. The area is characterized by a mixed type of farming systems. The climate alternates with long summer rainfall (June–September) and winter dry season (October–May) with mean annual rainfall [7]. The mean annual rainfall in the West Gojjam is 1352.9 millilitres. The cattle in the study areas are local indigenous zebu breed that are kept under traditional extensive husbandry systems with communal herding.

### 2.2. Study Design and Sampling Methods

A cross-sectional study was conducted from December 2017 to January 2018 to determine the relationship between trypanosomes infection rates and occurrence of anaemia and to estimate the prevalence of bovine trypanosomosis in West Gojjam zone, Northwest Ethiopia. Two districts, Debub Achefer and Semen Achefer, were purposively selected based on farmers' complaints of trypanosomosis problems. Then, the study sites were selected based on the accessibility of roads and the availability of sampling animals. Simple random sampling was employed to select individual study animals. The age of study animals was estimated based on dentition techniques given by De Lahunte and Habel [8] and information from owners. The sample size was determined considering 50% expected prevalence and 5% absolute desired precision at 95% confidence level based on the formula given by Thrusfield [9]. Accordingly, 384 study animals were needed; however, the sample from 730 cattle were collected, processed, and examined.

### 2.3. Blood Sampling and Examination

Paired blood samples were collected by puncturing the marginal ear vein of each animal into heparinized microhaematocrit capillary tubes (Deltalab SL, Barcelona, Spain). After sealing one end of capillary tubes with crystal sealant (Hawksley Ltd., Lancing, United Kingdom (UK)), samples were centrifuged using a microhematocrit centrifuge (Hawksley and Sons, UK) at 12000 revolutions per minute for 5 minutes. Anaemia was estimated by measuring PCV using the haematocrit reader. Animals with PCV less than 24% were considered anaemic, and the mean PCV between 24% and 48% was considered as normal threshold [10–12]. The contents of the capillary tubes including about 1 mm above and below the buffy coat were smeared on microscopic slides, covered with 22 ×× 22 cover slips, and examined under a ×40 objective of microscope using a dark ground buffy coat technique to detect the presence of motile trypanosomes. For positive samples, Giemsa stain of thin blood smears were made, fixed with methanol for 5 minutes, and examined under oil immersion using ×100 objective to identify the species of trypanosomes based on morphological characteristics [13].

### 2.4. Data Analysis

All statistical analyses were performed using STATA software version 12 (Stata Corporation, Texas, USA). The prevalence was calculated for all data as the number of infected individuals divided by the number of individuals examined and multiplied by 100. The association between trypanosome infection and anaemia (PCV <24%) was assessed by the chi-square test. The associations between the prevalence of trypanosomosis and associated risk factors such as districts, age groups, and sexes were assessed by the chi-square (*χ*^2^) test, whereas the *t*-test (two-group mean comparison test) was used to assess the difference in the mean PCV between trypanosome positive and negative animals, the trypanosome species, sex, and age groups. A statistically significant association between variables was said to exist if the *P* value is <0.05 at 95% confidence level.

## 3. Results

### 3.1. Parasitological Findings

The overall trypanosomosis prevalence was 7.81% and 95% CI = 7.45–8.17, in the studied area. Trypanosome infection was not associated with geographical district, age, or sex of the animals (Table 1).

### 3.2. Haematological Findings

The mean PCV of all examined cattle was 25.58 ± 3.95. The mean PCV in trypanosome positive (21.09%) and negative (25.96%) was significantly different (Table 2).

From the total *n* = 730 examined animals, 356 (48.77%) had a mean PCV below the reference interval (PCV <24%) and 374 (51.23%) had a mean PCV within the reference interval (24–48%); the infection was significantly associated with the anaemic state (Table 3). The mean PCV of cattle infected with *T. congolense* is lower than *T. vivax*. No difference in the PCV value was detected between male and female cattle and between young and adult cattle (Table 4).

## 4. Discussion

The study was conducted from December 2017 to January 2018 in West Gojjam zone, Northwest Ethiopia. The overall prevalence of bovine trypanosomosis in the studied area was 7.81% and 95% CI = 7.45–8.17. This value was in line with the reports of Abebe et al. [14] who reported trypanosome prevalence of 7.80% in Omo-Ghibe tsetse fly belt, South Ethiopia, and Tafese et al. [15] in East Wollega zone who reported trypanosomosis prevalence of 8.50%. The result of the current study was lower than a range of studies conducted previously in Ethiopia. Mekuria and Gadissa [16] reported 12.41% trypanosomosis prevalence in Metekel and Awi zones of Northwest Ethiopia and Afework et al. [17] in Metekel district reported 17.20%. Such variations may exist because of differences in agroclimates, distributions and density of vectors, season, and vectors control applications that may hinder the epidemiology of trypanosomosis.

Two *Trypanosoma* species, namely, *T. congolense* and *T. vivax* were identified in the studied area. These two trypanosomes species were also reported by Denbarga et al. [18] in the same studied area. Duguma et al. [19] and Cherinet et al. [20] also showed that these two trypanosomes species were encountered frequently in the country. *T. congolense* infection (4.25%) was higher than that of *T. vivax* (3.56%). This could be because the capacity of tsetse fly is probably more efficient in transmitting *T. congolense* than *T. vivax* [21]. Trypanosomosis prevalence was not significantly associated to districts, age, and sex of animals, which is in line with the report of Lelisa et al. [22] in West Ethiopia. Mulaw et al. [23] also described that there was no significant variation in trypanosomosis prevalence between sex and study sites in Northwest Ethiopia.

The mean PCV in trypanosome positive and negative cattle was 21.09 and 25.96%, respectively. Similar values were reported by different authors. Biyazen et al. [24] reported a mean PCV value of 22.36 and 27.86% in parasitaemic and aparasitaemic animals, respectively, in Western Ethiopia. Dagnachew et al. [25] reported mean 24.29% in trypanosome positive and 27.46% in Northwest Ethiopia. Desta et al. [26] also reported a mean PCV of 22.96% and 25.46% in trypanosome positive and negative, respectively. Degneh et al. [27] reported a mean PCV of 20.48% and 25.77% in positive and negative cattle. Waisma and Katunguka-Rwakishaya [28] reported 22.3% and 29.0% mean PCV in positive and negative cattle, respectively, in southwestern Uganda.

The mean PCV value in trypanosome-infected cattle was significantly lower than in the noninfected cattle population. Furthermore, as the development of anaemia is one of the cardinal signs of trypanosomosis [29], PCV decrease with the increasing prevalence of trypanosomosis is expected. The intensity of anaemia evidenced by declining PVC values was reported to be an indicator of the severity of trypanosomosis and correlates with the loss of production performance in susceptible or infected animals [6, 30–34].

However, there were anaemic cattle that were not infected with trypanosomes that would be due to clearance of the parasites from circulation with trypanocidal drugs and inadequacy of detection methods used (dark ground buffy coat technique). In addition, other factors, alone or in combinations, may induce anaemia in absence of trypanosomosis. Fasciolosis and gastrointestinal parasites that cause haemorrhagic anaemia, blood parasites [35, 36], and other diseases that cause erythrocyte haemolysis or specific nutritional deficiencies [37] may result in occurrence of anaemia.

The occurrence of positive animals with PCV of greater than 24% might be as a result of recent infection and may be due to animals that tolerated parasitaemia without showing anaemia [38]. There was no previous report on the multifactorial anaemia in the studied area, and their contribution cannot be ruled out. The current study indicated that PCV alone could not be utilized as a tool to diagnose trypanosomosis and agreed with the findings of Coetzer et al. [39] and Van den Bossche and Rowlands [40] who indicated measuring PCV could be not a confirmatory for diagnosis of trypanosomosis.

Both *T. congolense* and *T. vivax* were associated with lower PCV. Awa and Ndamkou [41] also showed that the mean PCV value was significantly affected by both *T. congolense* and *T. vivax*. PCV was significantly affected by trypanosome species. Cattle infected with *T. congolense* had a mean PCV lower than that infected with *T. vivax*. Sekini et al. [42] indicated that *T. congolense* is more pathogenic than *T. vivax* and cause severe parasitaemia and anaemia. In contrast to this finding, Achukwi and Musongong [43] showed that PCV is not affected by trypanosome species.

The occurrence of anaemia between the districts, age, and sex groups was not significant. This finding was in agreement with the report that revealed Ethiopian cattle breed of any sex and age did not show a significant difference in anaemia prevalence [44].

## 5. Conclusions

The prevalence of bovine trypanosomosis was 7.18%. Trypanosome infection and the anaemic state has a significant association Nevertheless, since there were animals that were positive for trypanosomes having mean PCV within the normal threshold and negative animals with anaemia, measuring PCV alone cannot be a confirmatory for diagnosis of trypanosomosis.

## Figures and Tables

**Table 1:** The prevalence of trypanosomosis and associated risk factors.

Risk factors	No. of examined (=*N*)	No. of positive	*χ* ^2^	*P* value
Districts				
Debub Achefer	420	36 (8.57%)	0.80	0.71
Semen Achefer	310	21 (6.77%)		

Age				
Young	372	27 (7.26%)	0.32	0.57
Adult	358	30 (8.38%)		

Sex				
Female	408	29 (7.11%)	0.63	0.43
Male	322	28 (8.70%)		

**Table 2:** The mean PCV of positive and negative cattle for trypanosomosis examined in West Gojjam, Northwest Ethiopia.

Group	Number examined	Mean	Standard error	Standard deviation	95% confidence level
Negative	673	25.96	0.14	3.73	25.68–26.24
Positive	57	21.09	0.48	3.63	20.13–22.05
Total	730	25.58	0.15	3.95	25.29–25.86

/*t*/ = 9.48; *P* ≤ 0.001; DF, 728; DF, degree of freedom.

**Table 3:** Association of trypanosomes infection and the occurrence of anaemia.

Status	Noninfected	Infected	Total	*x* ^2^	*P* value
Anaemic (PCV <24%)	307 (86.24%)	49 (13.76%)	356 (48.77%)	34.24	≤0.001
Nonanaemic (PCV ≥24%)	366 (97.86%)	8 (2.14%)	374 (51.23%)		
Total	673 (92.19%)	57 (7.81%)	730		

**Table 4:** Comparison of the mean PCV of infected cattle in association with the trypanosome species, sex, and age groups.

	Number of positives	Mean PCV (%)	SE	SD	95% CI	*P* value
Trypanosome species						
*T. congolense*	31	19.45	0.54	3.02	18.34–20.56	≤0.001
*T. vivax*	26	23.04	0.66	3.35	21.68–24.39	

Sex						
Male	28	20.57	0.74	3.93	19.05–22.06	0.29
Female	29	21.59	0.61	3.30	20.33–22.84	

Age						
Young	27	21.04	0.72	3.77	19.55–22.53	0.92
Adult	30	21.13	0.65	3.56	19.80–22.46	

CI, confidence interval; SD, standard deviation; SE, standard error.

## Data Availability

The datasets that support the findings of this study are available from the corresponding author upon request.
